# High-Resolution Mass Spectrometry Non-Targeted Detection of Per- and Polyfluoroalkyl Substances in Roe Deer (*Capreolus capreolus*)

**DOI:** 10.3390/molecules29030617

**Published:** 2024-01-27

**Authors:** Radmila Pavlovic, Susanna Draghi, Alberto Pellegrini, Claudia Fornesi Silva, Federica Di Cesare, Giulio Curone, Francesco Arioli, Marco Fidani

**Affiliations:** 1 Proteomics and Metabolomics Facility (ProMeFa), IRCCS San Raffaele Scientific Institute, Via Olgettina 60, 20132 Milan, Italy; pavlovic.radmila@hsr.it; 2 Department of Veterinary Medicine and Animal Sciences, University of Milan, Via dell’Università 6, 26900 Lodi, Italy; giulio.curone@unimi.it (G.C.); francesco.arioli@unimi.it (F.A.); 3 UNIRELAB Srl, Via Gramsci 70, 20019 Settimo Milanese, Italy; a.pellegrini@unirelab.it (A.P.); c.fornesi@unirelab.it (C.F.S.); m.fidani@unirelab.it (M.F.)

**Keywords:** high-resolution mass spectrometry, non-targeted analysis, compound discoverer, persistent organic pollutants, endocrine disruptors, biomonitoring, wild animals, ecotoxicology

## Abstract

Among wildlife species, roe deer stands out as a valuable indicator of environmental pollution due to its ecological significance and role as a game animal. The assessment of poly- and perfluoro substances (PFASs) bioaccumulation is of the utmost importance, relying on the liver and muscles as the main organs of interest. The study concerned the identification of 60 PFAS through a non-target workflow analysis based on HPLC Q-Exactive Orbitrap High-Resolution Mass Spectrometry in a homogeneous group of 18 female roe deer species. The developed strategy allowed us to individuate the 60 PFAS compounds with different levels of confirmation. Apart from seven PFASs identified via analytical standards, the remaining fifty-three features were identified with CL 2 or 3. Moreover, by applying a differential statistic approach, it was possible to distinguish the bioaccumulation patterns in the liver and muscle, identifying 12 PFAS upregulated in the muscle and 20 in the liver. The analysis reveals that specific PFAS compounds present exclusively in either the muscle or in the liver. The study emphasises the specificity of the liver and muscle as significant bioaccumulation sites for PFAS, raising questions about the underlying mechanisms of this process. In conclusion, the presented non-targeted PFAS analysis workflow evidenced promising and reliable results, successfully demonstrating its feasibility in the field of environmental research.

## 1. Introduction

Per- and polyfluoroalkyl substances (PFASs) are a group of synthetic chemicals that have been used extensively in industrial and consumer products for several decades due to their thermal and chemical stability, in addition to their amphiphilic nature [1,2,3]. The most frequently studied PFASs, namely perfluorooctanoic acid (PFOA) and perfluorooctane sulfonate (PFOS), are listed under the Stockholm Convention on Persistent Organic Pollutants ‘due to their demonstrated toxicity, bioaccumulation, persistence in the environment, and ability to travel long distances from the point of release or application [4]. Through different pathways, PFASs can enter the environment; moreover, these compounds do not readily degrade under natural conditions, with consequent bioaccumulation over time and increasing persistence within all aquatic and terrestrial ecosystems [5,6,7,8]. Evidence demonstrates that PFASs can have adverse effects on various biological processes, including immune function, reproductive health, and development [9].

Studies have detected PFASs in various wild animal species, including birds, fish, and mammals [10,11,12,13]. These chemicals have been found in the blood, liver, and muscle tissues of animals, indicating that they are absorbed and distributed throughout the body [11,14]. Top predator species, such as eagles, bears, and snakes, have been found to have higher levels of PFAS compared to lower trophic-level species, indicating that they are at a higher risk of exposure [15,16]. The specific pathways of PFAS exposure in wildlife can vary depending on the local environment, industrial activities, and land use practices. 

The European roe deer (*Capreolus capreolus*) is one of the wild mammals that has been identified as a good bioindicator due to its distinctive behavioural traits [17]. These include its small home ranges (16–80 ha) and high behavioural plasticity, which enable it to live in a variety of habitats, including those that are heavily used by humans [17,18]. The primary foods that roe deer eat include grass, leaves, berries, and young shoots. They are often adapted to eat readily digested forages, and certain research has shown that the number of pollutants in their muscles varies depending on what they eat [18,19].

As there is little information on the manufacturing and use history for most PFASs present on the market due to limited communication between the public and manufacturers, some unknown PFASs may be present in the environment and, thus, in living organisms, without any public alert in this regard. Therefore, the rapid progress in the chemical industry in terms of PFASs, the increasing evidence of their high toxicity, and the possibility of provoking metabolic alterations in biota necessitate an advanced analytical approach that would be able to monitor known PFAS, but contemporary to identify those that not considered/defined yet.

The emerging approach to evaluating PFASs is to implement complementary targeted and non-targeted analyses [6]. A wide range of methodologies have been presented to identify per- and polyfluoroalkyl substances (PFASs) in the environment [20], but the application of liquid chromatography in conjunction with high-resolution mass spectrometry (LC-HRMS) has unquestionably been crucial for expanding the scope of PFAS detection in environmental matrices [21]. 

Targeted analyses aim to quantify the occurrence of known PFASs by means of HPLC-HRMS in the pg/g range [10,11]. Non-targeted analyses aim to characterise PFASs more comprehensively, including PFASs that are unexpected or unknown, also by means of HPLC-HRMS but with the subsequent retrospective data processing performed with some of the available metabolomics software [22]. The general non-targeted approach relies on data-mining techniques to discover evidence of fluorinated compounds, which can be prioritised for structure elucidation. Several data-processing techniques have been described for non-targeted analysis of PFASs, including characteristic fragment ion searching and direct homologous series detection [9,22,23,24]. Although non-targeted analyses are inherently qualitative, efforts have been made to estimate concentrations of non-targeted PFASs using matching data from complementary target analyses [25].

Most non-targeted analysis has been performed either on environmental samples or on human blood, tissue and fluids, and just sporadically on the animal matrices and species [23]. For the last ones, the available literature deals almost exclusively with the classic targeted determination of known PFAS species [13,26]. As PFAS concentrations have been more frequently studied in aquatic food sources, there is less understanding of exposure in terrestrial animals.

In this scenario, considering that roe deer is a suitable bioindicator of the presence of these pollutants in the environment, the main goal of this research is to evaluate the presence and distribution of PFASs in roe deer liver and muscles, particularly those that would emerge from the non-targeted analysis. For this purpose, we have developed a non-targeted analysis based on liquid chromatography coupled with Q-Exactive Orbitrap high-resolution tandem mass spectrometry (HPLC-Q-Exactive Orbitrap-HRMS) applying Compound Discoverer 3.3 version software for PFAS identification and statistical evaluation.

## 2. Results and Discussion

### 2.1. Realisation of the Strategy for Non-Targeted PFAS Workflow

PFASs are an ever-present group of persistent toxic chemicals that have attracted the attention of legislative bodies worldwide [8]. Less than 200 PFAS standards are available for the more than 10,000 known PFASs, which highlights the requirement for a non-targeted approach in contrast to limited traditional target approaches. During the analytical and instrumental procedure setup, we first analysed the standard PFAS mixture (30 compounds, ISOmix, Chemical Research 2000 Srl, Rome, Italy) to evaluate the chromatographic and mass spectrometry method performance through retention times (RTs) and the isotopic and fragmentation patterns of the standard compounds that would serve for the evaluation of non-targeted results. The chromatographic behaviour of those compounds is presented in Figure S1a–d as extracted ion chromatograms of the parent ions [M-1]^−^ in negative full-scan acquisition mode. As can be noticed, we obtained sharp, narrow peaks for all compounds included with RTs associated with chain length and the presence of different functional groups. Additionally, running the standard mix helped in the high-confidence identification of some m/z signals that emerged from the untargeted analysis. 

Belonging to the same homologous series and having specific fragments with progressive RTs connected to chain length, negative mass defect (MD) and Kendrick MD are the main components of the non-targeted PFAS determination procedures [20]. Each of these methods is only partially efficient in finding PFASs, and only by merging them can a high level of identification confidence be achieved. This is achieved by the recently released Compound Discoverer 3.3 Untargeted PFAS workflow that consists of spectra selection, alignment of retention time, the precursor ions collection consulting Compound Discoverer integrated databases (https://www.mzcloud.org (accessed on 10 December 2023) and https://www.chemspider.com (accessed on 10 December 2023), and normalisation of the chromatographical peaks area. The output of the Compound Discoverer 3.3 consists of a feature list containing molecular formulas, accurate molecular mass, m/z value, intensity (peak area), retention time, and results of mass list matches. The initial output of our Compound Discoverer analysis contained more than 17,742 features that were grouped and filtered using various parameters to reduce data quantity and complexity.

Common practices for data reduction before PFAS-specific feature filtering include background signal removal. When PFASs are present at trace levels, it can be difficult to distinguish probable PFAS ions from background signals in full-scan spectra, which represents the most challenging and important phase in the non-target PFAS identification process. It is standard procedure in non-target discovery research to remove background signals from the equipment or reagents as the initial step. In our analytical workflow, it was carried out in three different manners: (1) using the additional CMB WR C18 column to delay the retention times of PFASs originating from the system, as can be noticed for FOSA (Figure S1d); (2) generating an exclusion list of features that emerged from the double-round run of procedural blanks; and (3) including background removal as a node in the Compound Discoverer workflow, where the full-scan HRSM data and prioritisation focused on features with a ratio below 10 compared to the background in any sample group. Additionally, the intensity threshold cutoff was set to 1,000,000 arbitrary units to exclude the low abundant peaks. Filtering according to the Kendrick MD (range of −0.25 to +0.15) was chosen as PFASs generate mostly negative mass defects due to the fact that they replace many hydrogen atoms with fluorine atoms. The Kendrick system converts masses from the IUPAC system based on 12C = 12.0000 to a mass referential based on CF2 = 50.0080 for the identification of homologue compounds, differing only by their number of CF2 moieties. In this manner, 373 PFAS candidates were isolated and subjected to final characterisation. 

Consequently, only the features for which an MS^2^ (data-dependent acquisition, DDA, for preferred ion) was available were selected for definitive confirmation. Finally, the main criteria for PFAS putative identification via the Compound Discoverer workflow were chosen as a combination of two different assets: a mzCloud match score higher than 60% and/or the identification being proposed by at least one integrated Mass List. The assessment of the fragment patterns (Table 1) was performed manually using ChemDraw 14.0 version software or consulting online in silico fragmentation tools CFM-ID and MetFrg [27,28]. 

While the MS/MS acquisition mode enables structural proposals for the unknown analyte, structural confirmation requires comparison to an authentic standard, which for some suggested PFAS was possible due to the runs of analytical standards’ mixture. However, structural suggestions relying solely on mass spectral evidence prove beneficial to the scientific community and are frequently documented in peer-reviewed literature for the identification of non-target PFAS compounds. Nevertheless, these suggestions should be coupled with statements acknowledging the presence of uncertainties. A useful guide that has been used for PFAS is the confidence levels recommended by Schymanski et al. (2014) [29] and detailed more profoundly by Nason et al. (2020) [22]. In this context, the confidence levels (CLs) span from 1 to 5, with 1 indicating the highest confidence level, supported by a validated standard; level 2 signifies likely structures based on comparisons with library spectra or diagnostic evidence; level 3 denotes potential candidates with identifiable structures but insufficient information for precise assignment; level 4 is assigned when the unknown analyte ion can be attributed only to an unambiguous formula; and level 5 represents the lowest confidence level, which is employed when proposing even a single empirical formula is uncertain. Most published non-target PFAS identifications are in the CL 2 e 3 range [13,25,30], as reliable standards are hardly available. Applying the above strategy, it was possible to individuate the list of 60 PFAS compounds with different levels of confirmation (Table 1). The putative identification was performed strictly by applying the feature filters explained in detail in the Material and Methods section. Apart from seven PFASs identified using analytical standards, the remaining fifty-three features were identified with CL 2 or 3 and were attributed to IUPAC name, formula, and the m/z of the parent ions and diagnostic ions. Other identification characteristics are present in Table S2, along with the proposed structures that are reported in Table S1.

Two compounds belonging to the class of perflorosulfonates (PFSA) were detected using the corresponding analytical standards, namely **50** (Perfluoro-n-hexanesulfonate) and **51** (Perfluoro-n-octanesulfonate). In the case of PFSAs, the relatively long chromatographic run applied herein with the well-separated features increased additional certainty and improved the identification approach. It is important to underline that by using the established workflow, it was possible to distinguish some structural isomers. Usually, retention times and fragmentation patterns are used to characterise individual isomers but may complicate the analysis and impact accuracy at low concentrations and when isomers have very similar chemical and physical properties [25]. For example, the chromatographic separation of two features with m/z = 498.9319 and 498.9321 (Figure S2) with retention times of 17.09 min and 18.26 min, respectively, revealed the presence of perfluoro-6-methylheptanesulfonate (**49**) and perfluoro-n-octanesulfonate (**51**). Species **49** had the shorter RT and resembled a branched carbon chain (6-trifluoromethyl isomer), while species **51** corresponded to a linear backbone and has been confirmed by using the analytical standard. 

Five perfluorocarboxilic acids (compounds **54**–**58**) were also detected with a confidence level of 1. Comparing the obtained non-target results with standard mix runs and the fragmentation patterns of targeted DDA MS/MS spectrums, it was noticed that in non-targeted DDA acquisition fragments, the output and ratio were influenced by the sample’s matrix. Therefore, the filtering of the minimum chromatographical peak area (greater than 1,000,000 arbitrary units), despite reducing the number of features detected, increased the reliability of the identified species.

### 2.2. Investigating the Presence of PFAS in Roe Deer Liver and Muscle

**Figure 1:**
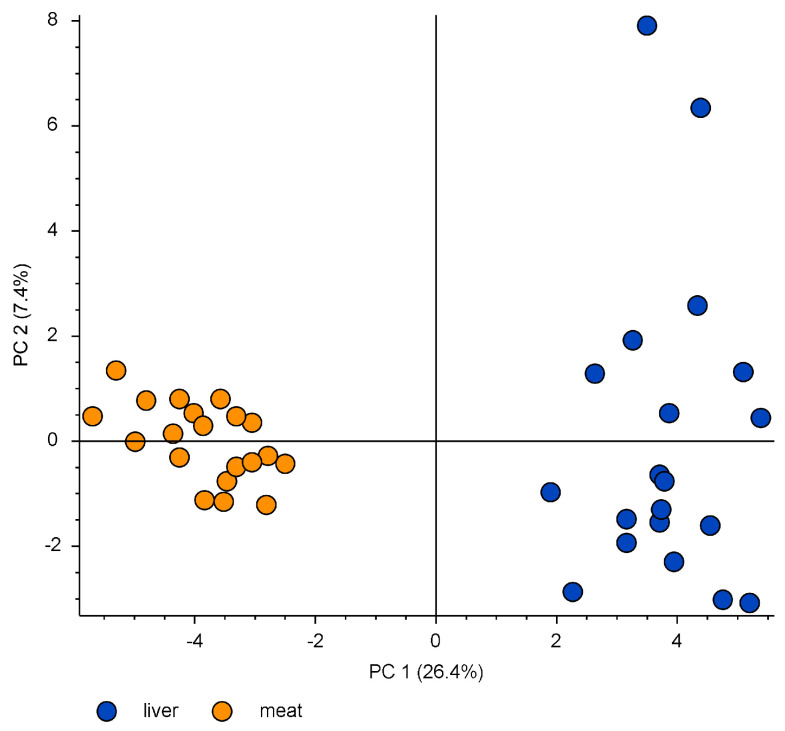
Principal Component Analysis (PCA) projection on the distribution of PFASs in liver and muscle roe deer samples.

Wildlife functions as a significant marker of environmental pollution, offering valuable information about the accumulation and spread of contaminants within ecosystems [15]. Roe deer is especially noteworthy among wildlife species, given its ecological importance and its status as a game animal frequently consumed by humans [17,26]. Assessing PFAS bioaccumulation is crucial, and the liver and muscles of roe deer play a pivotal role in this evaluation, serving as essential organs in metabolic processes and also being the tissues consumed by humans. In the initial step, taking into consideration 60 PFASs identified with the above-described non-target workflow, PCA evaluation was performed (Figure 1). The clear distinction between the two matrices is evident, indicating the independent bioaccumulating pattern concerning the liver and the muscle. 

**Figure 2:**
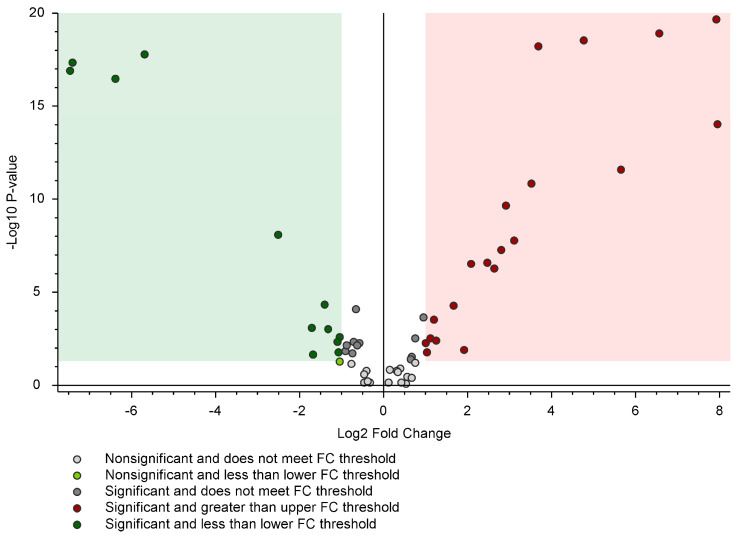
Volcano Plot comparison between the relative intensity of PFAS normalised chromatographic peak from liver and muscle roe deer samples. The green region contains downregulated PFASs, with intensities from the liver significantly lower than those from the muscle. The red region includes upregulated peaks where the intensities from the liver were significantly higher than those from the muscle. *p*-value (PV) = 0.05; Log2 Fold Change = 1.

While PCA was based on the first two principal variables, the Volcano Plot (VP) analysis was constructed on Log_2_ (Fold Change, FC) and -log_10_ (*p*-value) parameters in order to individuate the main differentiators (Figure 2). Of the initial 60 compounds, 12 PFASs were upregulated in the muscle (green region of VP), while 20 were upregulated in the liver (red VP area). All data regarding VP analysis with FC and *p*-values for all PFAS are presented in Table S2.

**Figure 3:**
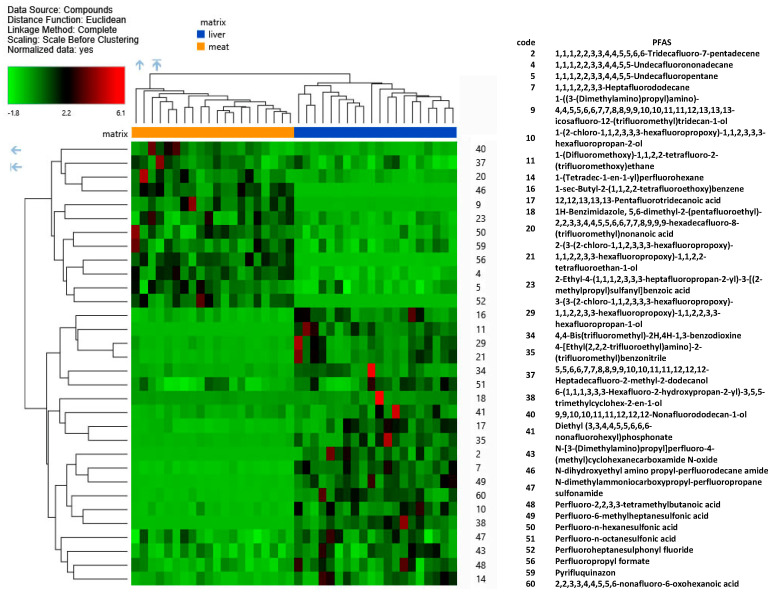
Hierarchical cluster analysis: heat map revealing the variations between PFASs discovered in HPLC-Q-Exactive-Orbitrap-MS non-target analysis. Only the compounds significantly different in the Volcano plot evaluation are viewed.

Lastly, Hierarchical Clustering (heat map) Analysis (HCA) was created to visually represent the behaviour of 32 statistically significant differential PFASs in the liver and muscle sample groups selected from VP evaluation. The map illustrates the intensities of each identified compound (Figure 3), with compounds showing significantly higher abundance depicted in red and those with lower abundance appearing in green. The compounds **2** (1,1,1,2,2,3,3,4,4,5,5,6,6-Tridecafluoro-7-pentadecene), **9** (1-((3-(Dimethylamino)propyl)amino)-4,4,5,5,6,6,7,7,8,8,9,9,10,10,11,11,12,13,13,13-icosafluoro-12-(trifluoromethyl)tridecan-1-ol), **11** (1-(Difluoromethoxy)-1,1,2,2-tetrafluoro-2-(trifluoromethoxy)ethane), and **38** (6-(1,1,1,3,3,3-Hexafluoro-2-hydroxypropan-2-yl)-3,5,5-trimethylcyclohex-2-en-1-ol) were exclusively detected in the muscle, while **46** (N-dihydroxyethyl amino propyl-perfluorodecane amide), **52** (Perfluoroheptanesulphonyl fluoride), and **56** (Perfluoropropyl formate) were present only in the liver matrix. A few individual samples demonstrated peculiarly high concentrations of some PFASs. For example, three liver samples with remarkably elevated amounts of **41** (Diethyl (3,3,4,4,5,5,6,6,6-nonafluorohexyl)phosphonate, **18** (1H-Benzimidazole,5,6-dimethyl-2-(pentafluoroethyl)-, **34** (4,4-Bis(trifluoromethyl)-2H,4H-1,3-benzodioxine, and **60** (2,2,3,3,4,4,5,5,6-nonafluoro-6-oxohexanoic acid), were found. The liver is commonly chosen for analysis because it tends to accumulate higher concentrations of PFASs compared to other organs. Monitoring PFAS levels in the liver of roe deer can provide insights into the extent of environmental contamination, especially for the exemplars where the untargeted PFAS screening has revealed particularly high concentrations. It has been demonstrated that the strong correlations between known PFAS contamination and PFAS patterns observed in the livers for each site suggest a source-specific accumulation of PFASs in wildlife livers [13]. The results that have emerged from this study emphasise that the muscle is an important bioaccumulation site for PFASs, but the mechanisms of this process remain to be elucidated.

## 3. Materials and Methods

### 3.1. Chemicals and Reagents

All the solvents used in this experiment: 25% ammonia solution, methanol for HPLA LC-MS grade and acetonitrile have been purchased from VWR International S.r.l. (Radnor, PA, USA). The ammonium formate and the native stock solution containing the standards (ISOmix, 30 compounds) were purchased from Chemical Research 2000 Srl (Rome, Italy). ISOmix contains the following PFAS: perfluoro-n-butanoic acid (PFBA), perfluoro-n-pentanoic acid (PFPeA), perfluoro-n-hexanoic acid (PFHxA), perfluoro-n-heptanoic acid (PFHpA), perfluoro-n-octanoic acid (PFOA), perfluoro-n-nonanoic acid (PFNA), perfluoro-n-decanoic acid (PFDA), perfluoro-n-undecanoic acid (PFUdA), perfluoro-n-dodecanoic acid (PFDoA), perfluoro-n-tridecanoic acid (PFTrDA), perfluoro-n-tetradecanoic acid (PFTeDA), perfluoro-n-hexadecanoic acid (PFHxDA), perfluoro-n-octadecanoic acid (PFODA), perfluoro-1-octanesulfonamide (FOSA), N-methylperfluoro-1-octanesulfonamide (N-MeFOSA), N-ethylperfluoro-1-octanesulfonamide (N-EtFOSA), N-methylperfluoro-1-octanesulfonamidoacetic acid (N-MeFOSAA), N-ethylperfluoro-1-octanesulfonamidoacetic acid (N-EtFOSAA), 2H-perfluoro-2-decenoic acid (FOUEA), 2,3,3,3-Tetrafluoro-2- (1,1,2,2,3,3,3-heptafluoroproproxy)propanoic acid Genx-NH3) (HFPO-DA), Potassium perfluoro-1-butanesulfonate (PFBS), sodium perfluoro-1-hexanesulfonate (PFHxS), sodium perfluoro-1-heptanesulfonate (PFHpS), sodium perfluoro-1-octanesulfonate (PFOS), sodium perfluoro-1-decanesulfonate (PFDS), sodium 1H,1H,2H,2H-perfluorooctanesulfonate (6:2FTS), sodium 1H,1H,2H,2H-perfluorodecanesulfonate (8:2FTS), sodium dodecafluoro-3H-4,8-dioxanonanoate (NaDONA), potassium 9-chlorohexadecafluoro-3-oxanonane-1-sulfonate (9Cl-PF3ONS), and sodium bis (1H,1H,2H,2H-perfluorodecyl) phosphate (8:2diPAP) at concentration of 100 ng/mL, each. Water was purified using a Milli-Q system (Millipore, Merck KGaA, Darmstadt, Germany). Before the beginning of the experiment, individual stock standard solutions of IS were prepared at the concentration of 1 ug/mL in methanol and stored at −20 °C. Working solutions were prepared daily via the dilution of the stock standard solutions in methanol.

### 3.2. Sample Collection and Extraction

The samples used in this study were female roe deer (in total 20 animals) originating from Oltrepò Pavese, Pavia province, Lombardy region, Italy, who were killed during regular hunting activity by authorised hunters. From each animal, 100 g of muscle (*Longissimum lumborum et thoracis*, on the left side of the carcass) and 100 g of liver were collected. The samples were placed into glass tubes, immediately refrigerated at 4 °C, transported to the laboratory and then frozen at −20 °C until further analyses according to an intralaboratory method already validated in different biological matrices [10,11] with some modifications. Briefly, 5 g of muscle or liver was transferred into a new glass tube, and 10 mL of acetonitrile was added for PFAS extraction and protein precipitation. Then, the samples were homogenised by using a T25 Digital ULTRA TURRAX^®^ for 1 min. To avoid cross-contamination, the blade of the homogeniser was washed with water, dried with paper, washed with 75% ethanol, and rinsed with water after each sample. After homogenisation, the samples were vortexed, sonicated for 15 min, centrifuged (2500× *g*, 4 °C, 10 min), and then the supernatant was transferred into a new glass tube and dried in a rotary vacuum centrifuge at 55 °C.

The dried extract was suspended in 10 mL of purified Milli-Q water and underwent SPE extraction using the Strata PFAS (WAX/GCB), 200 mg/50 mg/6 mL purchased from Phenomenex SRL (Torrance, CA, USA) under vacuum for further purification and extraction. The SPE cartridges were preconditioned with 4 mL of 0.3% ammonia in methanol, 4 mL of methanol, and 4 mL of Milli-Q water. The sample was loaded, and then the cartridges were washed with 2 × 4 mL of Milli-Q water, followed by 2 mL of methanol. Finally, the compounds were eluted using 2 × 4 mL of 0.3% ammonia in methanol and were collected in a 15 mL glass tube. The eluate was dried in a rotary vacuum centrifuge at 55 °C. At the complete drying, the samples were resuspended in 100 uL of MeOH + 100 uL mobile phase (90% water with ammonium formate 20 mM and 10% of MeOH), centrifuged for 2 min at 23,500× *g* and transferred into vials for HPLC Q-Exactive Orbitrap High-Resolution Mass Spectrometry analysis.

### 3.3. HPLC Q-Exactive Orbitrap High-Resolution Mass Spectrometry Analysis

The HPLC-HRMS system consists of a Vanquish (Thermo Fisher Scientific, Waltham, MA, USA) (equipped with a binary pump, auto-sampler and thermostat compartment for two columns) coupled to a Thermo Q Exactive Orbitrap^TM^ (Thermo Fisher Scientific), equipped with a heated electrospray ionisation source. Chromatographical separation was achieved using a Raptor ARC-18 5 um 150 × 2.1 mm column equipped with a Raptor ARC-18 5 um EXP guard column (Restek, Bellefonte, PA, USA). Furthermore, an additional column, CMB WR C18 50 mm × 4.6 mm, 10 um (Perkin Elmer, Waltham, MA, USA), was installed in front of the injector to delay the eventual PFASs already present in the system. The mobile phase consisted of phase A (20 mM aqueous ammonium formate) and phase B (MeOH). The gradient started with 20% of phase B, which reached 95% of B at the 7th min and was kept in this condition for 3 min. At the 11th min, the initial conditions (20% B) were reached and kept for 4 min for requilibration. The total run time was 15 min. The run was performed at 0.3 mL/min. For the detector parameters, capillary and vaporiser temperatures were set at 330 and 280 °C, respectively, the sheath and auxiliary gas at 35 and 15 arbitrary units, and the electrospray voltage at 3.50 kV, operating in the negative mode. The full scan (FS) acquisition was combined with a DDA, including an exclusive list prepared based on the features that appeared in the procedural blank. The FS worked with a resolution of 70,000 FWHM, a scan range of 200–950 m/z, an automatic gain control (AGC) of 1E6, and a maximum injection time of 200 ms. The DDA acquisition operated at 35,000 FWHM, with an AGC target of 5 × 10^4^, a maximum injection time of 100 ms, and an isolation window of 2 m/z. The fragmentation of the precursors was optimised with a two-step normalised collision energy (10 and 70 eV). Xcalibur^TM^ 4.3 (Thermo Fisher Scientific) was used.

### 3.4. Non-Targeted PFAS Workflow: Find and Identify Per- and Polyfluoroalkyl Substances

The raw data from Q Exactive Orbitrap analysis were elaborated using Compound Discoverer (CD) 3.3 software (demo version, Thermo Fisher, MA, USA), which permitted the programmed PFAS annotation and statistical evaluation. The standard CD non-targeted PFAS workflow was used to perform retention time alignment, unknown compound detection, and compound grouping across all samples through the standard CD workflow nodes. As a part of standard CD data processing, this workflow identifies compounds using mzCloud (ddMS2) and ChemSpider (formula or exact mass), removing the background signals utilising the procedural blank samples [31]. Furthermore, this PFAS CD pipeline indicates compounds that match with specific PFAS mass lists through the Search Mass Lists node that includes the following: PFASSTRUCT-2022-04-20 available on https://comptox.epa.gov/dashboard/chemical-lists/PFASSTRUCT (accessed on 10 December 2023), PFASNEG adapted from Barzen-Hanson et al. (2017) [30], and PFASNIST referenced as a suspect PFAS list published on the National Institute of Standards and Technology (NIST) science data portal (https://data.nist.gov/od/id/mds2-2387 (accessed on 10 December 2023)) [32]. The Compound Class Scoring node indicates the PFASs that share a common set of fragments. The Calculate Mass defect node reveals the defect values based on the selected mass defect type (Kendrick for identifying homologous series). Finally, to guarantee sufficient permanence of the sequence analysis, each sample was subjected to the workflow twice, and quality controls (QC) were applied at random throughout the analytical batch. For every experiment, the identical volume of the authentic samples was combined to create the QC samples. Additionally, each batch included a procedural blank sample to detect the background signals.

### 3.5. Statistical Evaluation

Descriptive, univariate, and multivariate statistical analyses were performed as an integral part of the CD workflow throughout the Differential Analysis node. It consisted of Principal Component Analysis (PCA), Hierarchical Clustering (heat map) Analysis (HCA), and Volcano plot (VP) processing.

## 4. Conclusions

In conclusion, the bioaccumulation of PFASs in the muscles and livers of roe deer raises concerns and could have ecological implications, considering that there is very limited information available regarding their toxicity and persistence in the environment. Monitoring and understanding bioaccumulation are essential for assessing the health of ecosystems and making informed decisions regarding environmental management and conservation. In this scenario, the development of statistical and analytical non-targeted platforms in order to expand the use of non-targeted data beyond a single study is one problem facing PFAS exposomic research at the moment. The presented analysis workflow allowed us to successfully individuate 60 PFAS compounds with a consistent rate of features detected at very high levels of confirmation. Moreover, by applying this strategy, it was possible to distinguish the bioaccumulation patterns in the different evaluated matrices, identifying 12 PFASs upregulated in the muscle and 20 in the liver. In conclusion, the developed non-targeted PFAS analysis workflow evidenced promising and reliable results, successfully demonstrating its feasibility in the field of environmental research. Additionally, the non-targeted measurements performed herein could have potential, especially if they could be harmonised with targeted data even in the absence of a priori technique standardisation.

## Figures and Tables

**Table 1:** List of the sixty PFAS tentatively detected in the liver and muscles of roe deer following the application of the non-targeted data analysis workflow.

Code	IUPAC Name	Formula	m/z [M-H]^−^	Mass Error [ppm]	Diagnostic Ions in DDA	RT [min]	Area (Max.)	Conf Level
**1**	1,1,1,2,2,3,3,4-Octafluoro-7,7-dimethyl-4-octene	C_10_H_12_F_8_	283.0731	−1.46	71.012; 115.247; 169.001	12.5	2.7 × 10^6^	3
**2**	1,1,1,2,2,3,3,4,4,5,5,6,6-Tridecafluoro-7-pentadecene	C_15_H_17_F_13_	443.1031	−4.2	85.011; 318.98; 343.976	12.64	3.9 × 10^7^	3
**3**	1,1,1,2,2,3,3,4,4,5,5,6,6-Tridecafluorohexadecane	C_16_H_21_F_13_	459.1341	−4.67	141.204; 318.979; 331.100	13.62	2.5 × 10^6^	2
**4**	1,1,1,2,2,3,3,4,4,5,5-Undecafluorononadecane	C_19_H_29_F_11_	465.2001	−4.27	181.201; 268.974	8.19	7.2 × 10^6^	3
**5**	1,1,1,2,2,3,3,4,4,5,5-Undecafluoropentane	C_5_HF_11_	268.9836	2.19	118.978; 169.001	13.61	4.3 × 10^7^	2
**6**	1,1,1,2,2,3,3,4,4-Nonafluorodocosane	C_22_H_37_F_9_	471.2660	−3.89	71.089; 387.1833	10.40	4.9 × 10^7^	3
**7**	1,1,1,2,2,3,3-Heptafluorododecane	C_12_H_19_F_7_	295.1306	1.34	275.128; 168.987	9.74	4.1 × 10^8^	3
**8**	1,1,1,3,3,3-Hexafluoro-2-(trifluoromethyl)propan-2-yl heptafluorobutanoate	C_8_F_16_O_2_	430.9587	3.86	118.96; 212.978; 218.78	8.46	4.9 × 10^6^	2
**9**	1-((3-(Dimethylamino)propyl)amino)-4,4,5,5,6,6,7,7,8,8,9,9,10,10,11,11,12,13,13,13-icosafluoro-12-(trifluoromethyl)tridecan-1-ol	C_19_H_19_F_23_N_2_O	727.1064	0.88	115.087; 129.112; 568.963; 695.043	11.65	1.0 × 10^7^	3
**10**	1-(2-chloro-1,1,2,3,3,3-hexafluoropropoxy)-1,1,2,3,3,3-hexafluoropropan-2-ol	C_6_HClF_12_O_2_	366.9409	2.15	116.996; 166.995; 232.987; 348.949; 366.944	17.86	7.4 × 10^7^	2
**11**	1-(Difluoromethoxy)-1,1,2,2-tetrafluoro-2-(trifluoromethoxy)ethane	C_4_HF_9_O_2_	250.9767	2.74	84.981; 134.986; 184.984	18.77	3.4 × 10^7^	2
**12**	(Pentafluoroethyl)-(trifluoromethyl)cyclohexane	C_9_H_10_F_8_	269.0577	−2.04	118.989; 151.097; 201.091	11.82	3.9 × 10^8^	2
**13**	1-(Perfluoro-n-hexyl)dodecane	C_18_H_25_F_13_	487.1654	−4.16	318.979	12.46	3.1 × 10^7^	3
**14**	1-(Tetradec-1-en-1-yl)perfluorohexane	C_20_H_27_F_13_	513.1809	−4.21	318.979; 455.117; 493.178	13.63	3.0 × 10^7^	2
**15**	1-hydro-pentadecafluoroheptane	C_7_HF_15_	368.9772	1.64	168.987; 268.982	16.78	1.6 × 10^7^	2
**16**	1-sec-Butyl-2-(1,1,2,2-tetrafluoroethoxy)benzene	C_12_H_14_F_4_O	249.0904	−1.49	116.996; 133.065; 219.043; 221.061; 233.061	12.98	4.5 × 10^7^	3
**17**	12,12,13,13,13-Pentafluorotridecanoic acid	C_13_H_21_F_5_O_2_	303.1390	0.19	175.058; 213.071; 283.138	9.18	7.9 × 10^7^	3
**18**	1H-Benzimidazole, 5,6-dimethyl-2-(pentafluoroethyl)-	C_11_H_9_F_5_N_2_	263.0600	−4.8	118.069; 243.051	6.85	4.5 × 10^8^	2
**19**	1H-Perfluorohexane	C_6_HF_13_	318.9804	1.89	101.056; 118.987; 168.1987	15.65	3.0 × 10^7^	2
**20**	2,2,3,3,4,4,5,5,6,6,7,7,8,9,9,9-hexadecafluoro-8-(trifluoromethyl)nonanoic acid	C_10_HF_19_O_2_	512.9610	1.89	168.987; 344.987	18.29	1.6 × 10^7^	2
**21**	2-(3-(2-chloro-1,1,2,3,3,3-hexafluoropropoxy)-1,1,2,2,3,3-hexafluoropropoxy)-1,1,2,2-tetrafluoroethan-1-ol	C_8_HClF_16_O_3_	482.9300	2.8	67.001; 96.991; 116.998; 182.989; 298.977	19.76	9.9 × 10^7^	3
**22**	2-(Nonafluorobutyl)benzoic acid	C_11_H_5_F_9_O_2_	339.0085	3.52	295.0189; 339.007	10.22	3.2 × 10^7^	3
**23**	2-Ethyl-4-(1,1,1,2,3,3,3-heptafluoropropan-2-yl)-3-[(2-methylpropyl)sulfanyl]benzoic acid	C_16_H_17_F_7_O_2_S	405.0771	1.49	89.055; 207.028; 281.044	9.09	4.5 × 10^6^	3
**24**	2-Vinylperfluorobutane	C_6_H_3_F_9_	245.0030	4.71	224.998	8.97	1.8 × 10^7^	2
**25**	2H-Nonafluorobutane	C_4_HF_9_	218.9863	0.38	101.977; 118.997; 151.087	9.94	2.1 × 10^7^	2
**26**	3,4,5,5,6,6,7,7,8,8,9,9,10,10,10-Pentadecafluoro-3-decen-2-one	C_10_H_3_F_15_O	422.9880	2.01	380.9765; 404.9765	18.3	3.9 × 10^8^	2
**27**	3-((2-chloro-1,1,2,3,3,3-hexafluoropropoxy)difluoromethoxy)-1,1,2,2,3,3-hexafluoropropan-1-ol	C_7_HClF_14_O_3_	432.9323	1.25	67.0001; 116.997; 166.993; 182.999; 232.977	18.89	4.2 × 10^7^	3
**28**	3-(12,12,13,13,14,14,15,15,15-Nonafluoropentadecyl)-1,2-benzenediol	C_21_H_27_F_9_O_2_	481.1809	2.94	215.063; 323.100; 389.072	23.27	3.6 × 10^6^	3
**29**	3-(3-(2-chloro-1,1,2,3,3,3-hexafluoropropoxy)-1,1,2,2,3,3-hexafluoropropoxy)-1,1,2,2,3,3-hexafluoropropan-1-ol	C_9_HClF_18_O_3_	532.9271	3.08	182.99; 466.933	20.41	2.4 × 10^8^	3
**30**	3-(Butylsulfanyl)-2-ethyl-4-(1,1,1,2,3,3,3-heptafluoropropan-2-yl)benzoic acid	C_16_H_17_F_7_O_2_S	405.0771	1.54	181.033; 235.0798; 305.024; 315.026; 361.086	9.43	3.1 × 10^6^	2
**31**	3-[Ethyl(perfluoro-1-oxopentyl)amino]-2-hydroxypropyl heptanoate	C_17_H_24_F_9_NO_4_	476.1503	2.9	109.057; 218.986; 304.039; 346.095; 364.061; 448.115	11.79	3.2E × 10^6^	2
**32**	3-Fluoro-4-{(E)-[4’-(heptafluoropropyl)-4-biphenylyl]diazenyl}phenol	C_21_H_12_F_8_N_2_O	459.0763	2.93	109.012; 373.099; 439.069	13.73	5.6 × 10^7^	3
**33**	3-Pyridinecarboxamide, N-[4-(nonafluorobutoxy)phenyl]-	C_16_H_9_F_9_N_2_O_2_	431.0450	0.66	78.034; 104.014; 121.041; 181.041; 403.013; 431.044	10.87	5.6 × 10^6^	2
**34**	4,4-Bis(trifluoromethyl)-2H,4H-1,3-benzodioxine	C_10_H_6_F_6_O_2_	271.0192	−2.65	245.004	10.20	5.0 × 10^8^	3
**35**	4-[Ethyl(2,2,2-trifluoroethyl)amino]-2-(trifluoromethyl)benzonitrile	C_12_H_10_F_6_N_2_	295.0685	3.3	168.989; 211.995; 293.053; 295.068	7.61	5.7 × 10^7^	2
**36**	5,5,6,6,7,7,8,8,9,9,10,10,10-Tridecafluoro-1-decanol	C_10_H_9_F_13_O	427.0153	3.5	361.026; 391.0397	15.06	2.7 × 10^6^	3
**37**	5,5,6,6,7,7,8,8,9,9,10,10,11,11,12,12,12-Heptadecafluoro-2-methyl-2-dodecanol	C_13_H_11_F_17_O	505.0463	−0.63	485.040	9.03	9.7 × 10^6^	3
**38**	6-(1,1,1,3,3,3-Hexafluoro-2-hydroxypropan-2-yl)-3,5,5-trimethylcyclohex-2-en-1-ol	C_12_H_16_F_6_O_2_	305.0974	−2.66	123.087; 166.997; 287.087; 303.082;	13.55	2.7 × 10^7^	2
**39**	6-Chloro-1,1,2,2,3,3,4,4,5,5,6,7-dodecafluoroheptane	C_7_H_3_ClF_12_	348.9671	1.70	328.9596; 348.966;	9.59	6.8 × 10^7^	3
**40**	9,9,10,10,11,11,12,12,12-Nonafluorododecan-1-ol	C_12_H_17_F_9_O	347.1080	4.86	218.986; 273.033; 327.101	9.37	3.2 × 10^7^	2
**41**	Diethyl (3,3,4,4,5,5,6,6,6-nonafluorohexyl)phosphonate	C_10_H_14_F_9_O_3_P	383.0449	−4.03	106.991; 326.943; 337.075; 355.067	9.26	2.5 × 10^7^	2
**42**	Hexadecyl 2,3,3,3-tetrafluoropropanoate	C_19_H_34_F_4_O_2_	369.2405	−4.69	129.128; 144.991; 185.023; 349.236	11.41	2.1 × 10^7^	3
**43**	N-[3-(Dimethylamino)propyl]perfluoro-4-(methyl)cyclohexanecarboxamide N-oxide	C_13_H_13_F_13_N_2_O_2_	475.0713	3.4	115.087; 117.1033; 143.085; 145.098	12.14	8.7 × 10^6^	3
**44**	N-[3-(Dimethylamino)propyl]perfluorobutanamide	C_9_H_13_F_7_N_2_O	297.0843	0.02	168.992; 211.995; 240.026; 252.027; 277.078	11.92	1.5 × 10^8^	2
**45**	N-[3-(Dimethyloxidoamino)propyl]perfluoro-3,7-dioxaoctanamide	C_11_H_13_F_11_N_2_O_4_	445.0608	−4.23	115.092; 143.089; 211.087; 359.067; 384.014; 429.037	10.86	4.6 × 10^7^	2
**46**	*N*-dihydroxyethyl amino propyl-perfluorodecane amide	C_17_H_17_F_19_N_2_O_3_	657.0890	4.16	169.098; 468.977; 511.978; 554.0229; 613.147; 637.097	12.49	6.8 × 10^6^	2
**47**	N-dimethylammoniocarboxypropyl-perfluoropropane sulfonamide	C_9_H_13_F_7_N_2_O_4_S	377.0405	−1.84	143.083; 168.986; 232.951; 275.993; 315.004; 333.051	13.80	9.2 × 10^6^	2
**48**	Perfluoro-2,2,3,3-tetramethyl butanoic acid	C_8_HF_15_O_2_	412.9674	2.24	193.997; 368.978	16.79	2.4 × 10^7^	3
**49**	Perfluoro-6-methylheptanesulfonate	C_8_HF_17_O_3_S	498.9319	3.35	168.954; 330.158	17.91	8.7 × 10^7^	2
**50**	Perfluoro-n-hexanesulfonate	C_6_HF_13_O_3_S	398.9372	1.38	118.472, 168.278	15.75	6.3 × 10^7^	1
**51**	Perfluoro-n-octanesulfonate	C_8_HF_17_O_3_S	498.9321	3.77	118.232, 168.145; 218.784	18.26	1.5 × 10^8^	1
**52**	Perfluoroheptanesulphonyl fluoride	C_7_F_16_O_2_S	450.9282	−2.02	82.960; 368.980; 182.970	11.66	1.0 × 10^7^	2
**53**	Perfluoro-n-heptanoic acid	C_7_HF_13_O_2_	362.9704	2.27	96.974; 318.978	15.65	3.2 × 10^7^	1
**54**	Perfluoro-n-hexanoic acid	C_6_HF_11_O_2_	312.9735	2.3	193.97; 218.941; 268.983	13.61	3.2 × 10^7^	1
**55**	Perfluoro-n-nonanoic acid	C_9_HF_17_O_2_	462.9648	3.46	118.997; 298.048; 318.974	18.28	3.3 × 10^7^	1
**56**	Perfluoropropyl formate	C_4_HF_7_O_2_	212.9790	−0.9	94.990; 184.984	4.53	3.4 × 10^6^	2
**57**	Perfluoro-n-pentanoic acid	C_5_HF_9_O_2_	262.9766	2.15	118.950; 143.910; 218.990	9.94	1.9 × 10^7^	1
**58**	Perfluoro-n-unidecanoic acid	C_11_HF_21_O_2_	562.9583	2.63	116.97; 243.97; 318.89	20.21	2.0 × 10^7^	1
**59**	Pyrifluquinazon	C_19_H_15_F_7_N_4_O_2_	463.1013	0.55	103.030; 298.047; 313.024; 443.097	15.49	7.8 × 10^6^	3
**60**	2,2,3,3,4,4,5,5,6-nonafluoro-6-oxohexanoic acid	C_6_HF_9_O_3_	290.9723	4.87	168.972; 246.971	7.28	3.9 × 10^6^	3

## Data Availability

Data are contained within the article and Supplementary Materials.

## Supplementary Materials

The following supporting information can be downloaded at: https://www.mdpi.com/article/10.3390/molecules29030617/s1, Figure S1: Extracted ion chromatograms; Figure S2: Perfluoro-6-methylheptanesulfonate and perfluoro-n-octanesulfonate; Table S1: Proposed structures for tentatively identified compounds; Table S2: Compound Discoverer output.
