# Supplementary material for: High-Resolution Mass Spectrometry Non-Targeted Detection of Per- and Polyfluoroalkyl Substances in Roe Deer (Capreolus capreolus)
Source: Molecules. 2024 Jan 27;29(3):617. doi: 10.3390/molecules29030617 (PMC10856453; doi:10.3390/molecules29030617)
Supplement: Supplementary file 1 [file molecules-29-00617-s001.zip › Figure S1_a_b_c_d_Extracted ion chromatograms.pdf]

Supplementary Material

Figure S1. Extracted ion chromatograms of parent ions [M-H]$^-$ in negative full–scan acquisition mode for the PFAS from the standards mixture.

(a)

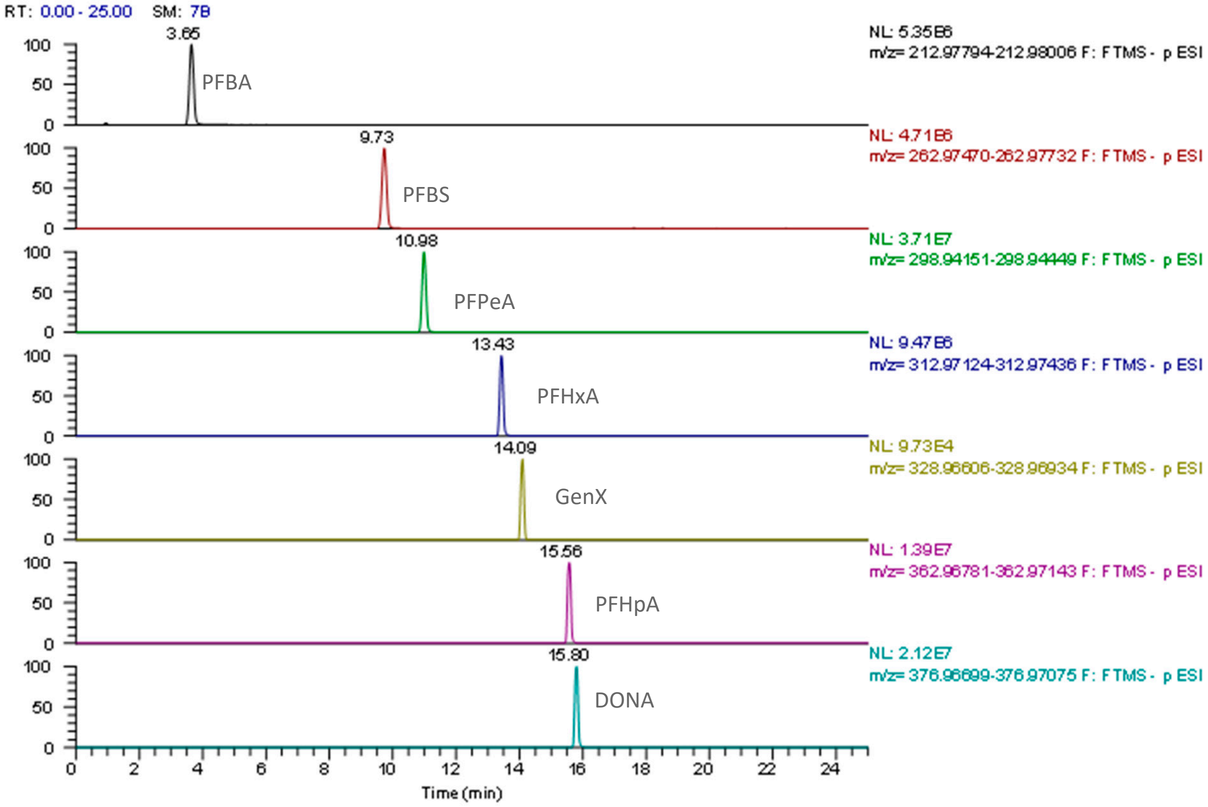

perfluoro-n-butanoic acid (PFBA), perfluoro-1-butanesulfonate (PFBS), perfluoro-n-hexanoic acid (PFHxA), perfluoro-n-pentanoic acid (PFPeA), 2,3,3,3-Tetrafluoro-2- (1,1,2,2,3,3,3-heptafluoroproproxy)propanoic acid Genx-NH3) (HFPO-DA), perfluoro-n-heptanoic acid (PFHpA), sodium dodecafluoro-3H-4,8-dioxanonanoate (DONA)

Figure S1 (continued). Extracted ion chromatograms of parent ions [M-H]$^-$ in negative full–scan acquisition mode for the PFAS from the standards mixture.

(b)

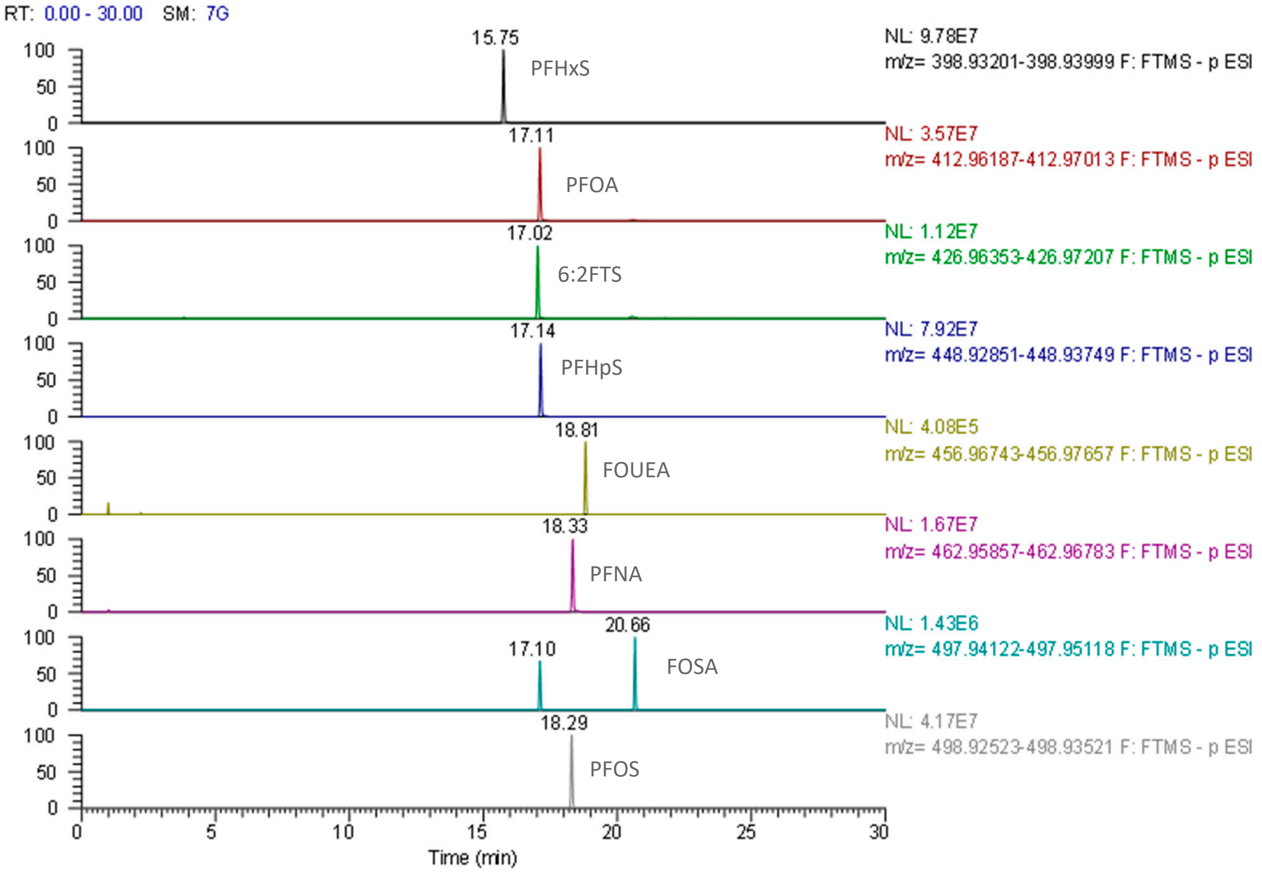

perfluoro-1-hexanesulfonate (PFHxS), perfluoro-n-octanoic acid (PFOA), 1H,1H,2H,2H-perfluorooctanesulfonate (6:2FTS), perfluoro-1-heptanesulfonate (PFHpS), 2H-perfluoro-2-decenoic acid (FOUEA), perfluoro-n-nonanoic acid (PFNA), perfluoro-1-octanesulfonamide (FOSA), perfluoro-1-octanesulfonate (PFOS)

Figure S1 (continued). Extracted ion chromatograms of parent ions [M-H]$^{-}$ in negative full–scan acquisition mode for the PFAS from the standards mixture.

(c)

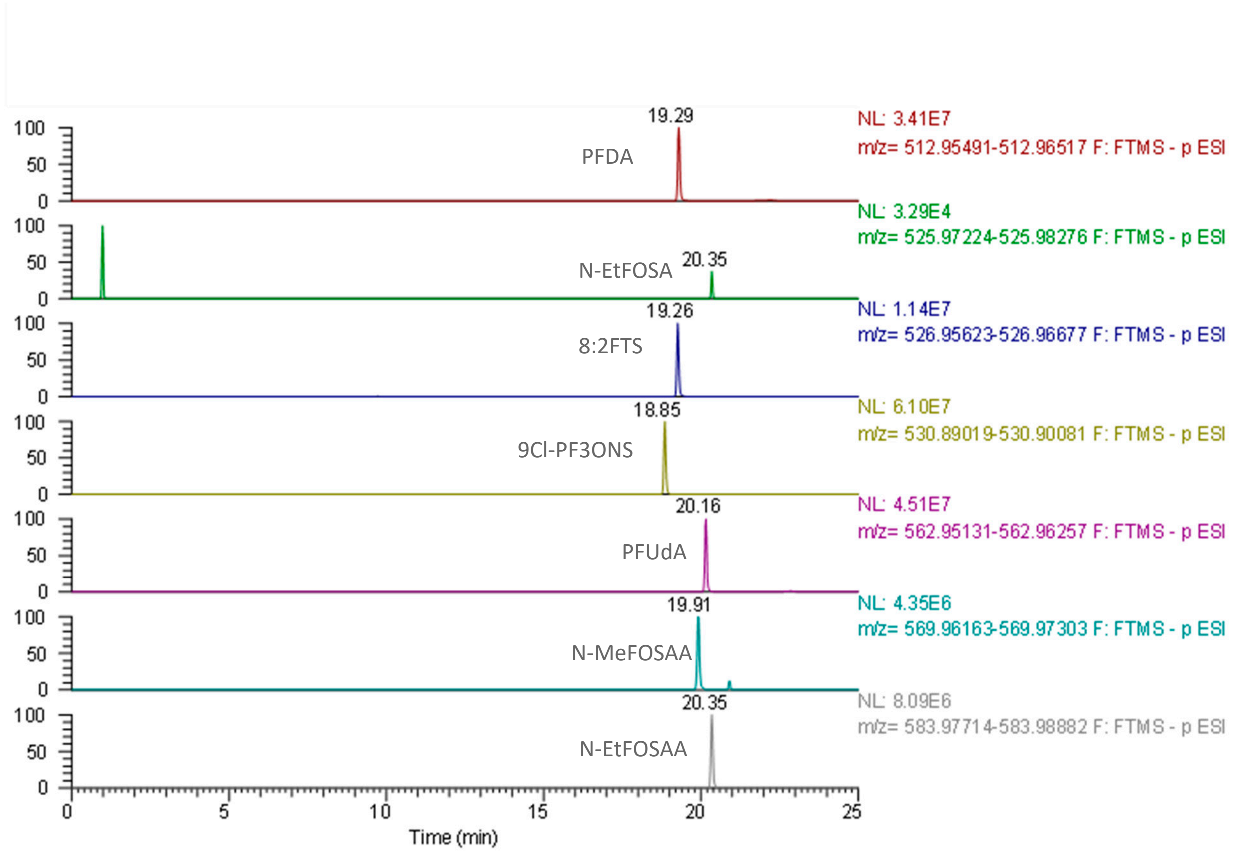

perfluoro-n-decanoic acid (PFDA), N-ethylperfluoro-1-octanesulfonamide (N-EtFOSA), 1H,1H,2H,2H-perfluorodecanesulfonate (8:2FTS), 9-chlorohexadecafluoro-3-oxanonane-1-sulfonate, perfluoro-n-undecanoic acid (PFUdA), N-methylperfluoro-1-octanesulfonamide (N-MeFOSA), N-methylperfluoro-1-octanesulfonamidoacetic acid (N-MeFOSAA), N-ethylperfluoro-1-octanesulfonamidoacetic acid (N-EtFOSAA)

Figure S1 (continued). Extracted ion chromatograms of parent ions [M-H]$^-$ in negative full–scan acquisition mode for the PFAS from the standards mixture.

(d)

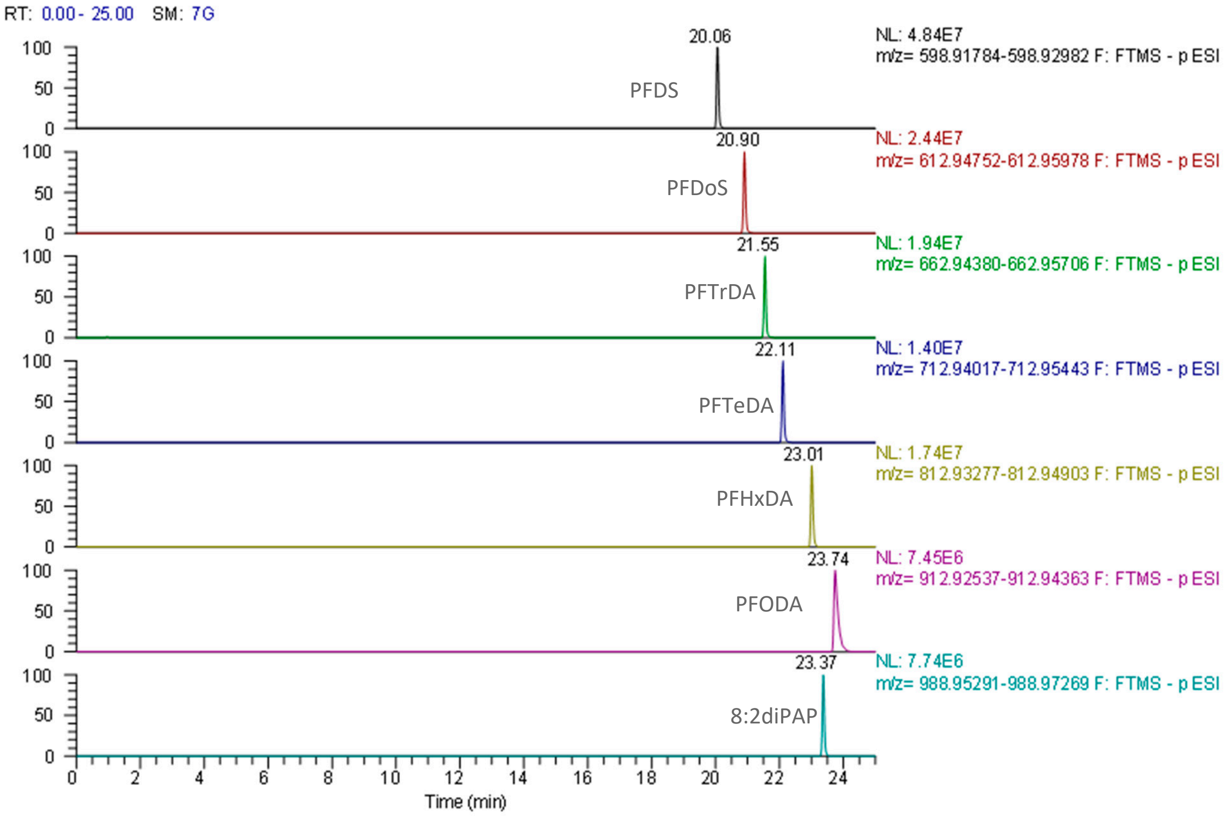

perfluoro-1-decanesulfonate (PFDS), perfluoro-n-dodecanoic acid (PFDoA), perfluoro-n-tridecanoic acid (PFTrDA), perfluoro-n-tetradecanoic acid (PFTeDA), perfluoro-n-hexadecanoic acid (PFHxDA), perfluoro-n-octadecanoic acid (PFODA), bis (1H,1H,2H,2H-perfluorodecyl) phosphate (8:2diPAP)
