# Supplementary material for: High-Resolution Mass Spectrometry Non-Targeted Detection of Per- and Polyfluoroalkyl Substances in Roe Deer (Capreolus capreolus)
Source: Molecules. 2024 Jan 27;29(3):617. doi: 10.3390/molecules29030617 (PMC10856453; doi:10.3390/molecules29030617)
Supplement: Supplementary file 1 [file molecules-29-00617-s001.zip › Figure S2_perfluoro-6-methylheptanesulfonate and perfluoro-n-octanesulfonate.pdf]

Supplementary Material

Figure S2. (a) Chromatographic separation of two structural isomers identified as perfluoro-6-methylheptanesulfonate and perfluoro-n-octanesulfonate (b) relative intensities of perfluoro-6-methylheptanesulfonate (c) relative intensities of perfluoro-n-octanesulfonate

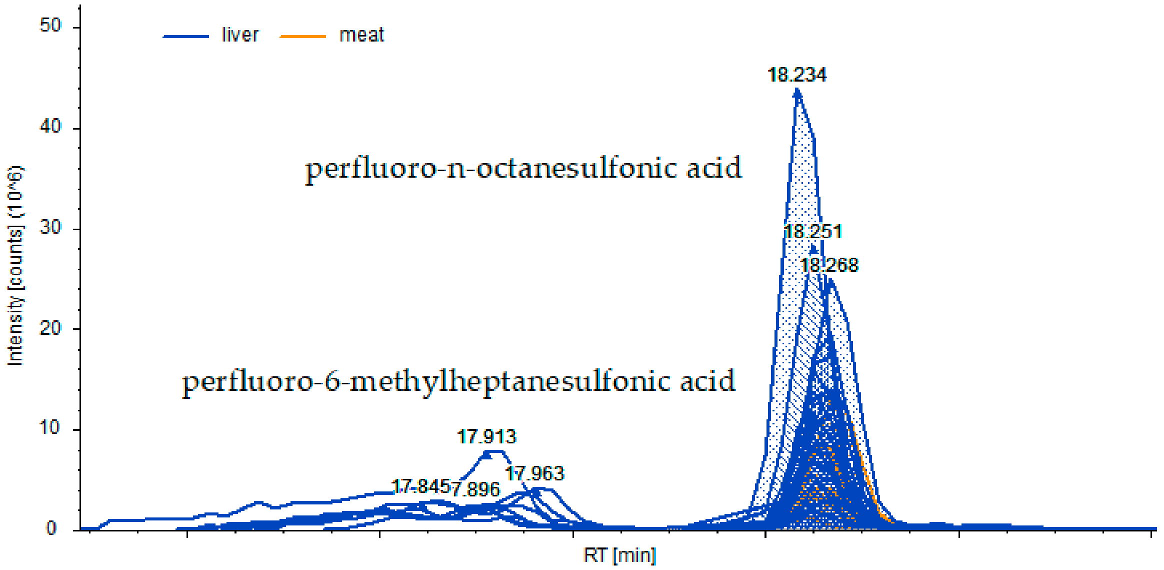

(a)

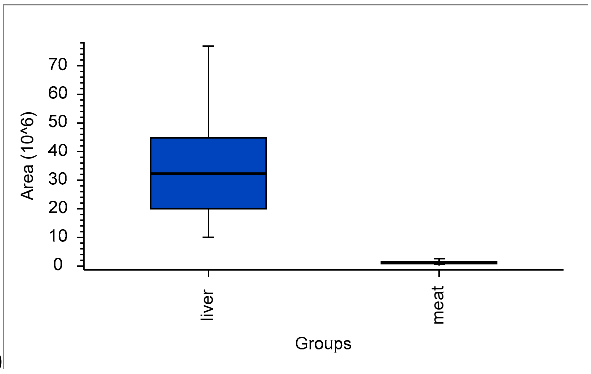

(b)

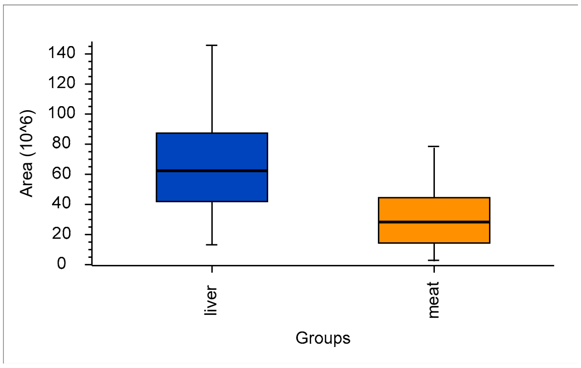

(c)
