# Supplementary material for: High-Resolution Mass Spectrometry Non-Targeted Detection of Per- and Polyfluoroalkyl Substances in Roe Deer (Capreolus capreolus)
Source: Molecules. 2024 Jan 27;29(3):617. doi: 10.3390/molecules29030617 (PMC10856453; doi:10.3390/molecules29030617)
Supplement: Supplementary file 1 [file molecules-29-00617-s001.zip › Table S1_Proposed structures for tentatively identified compounds.pdf]

Supplementary Material

Table S1. Proposed structures for tentatively identified PFAS.

**1**	1,1,1,2,2,3,3,4-Octafluoro-7,7-dimethyl-4-octene	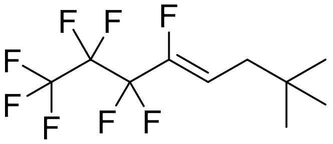
**2**	1,1,1,2,2,3,3,4,4,5,5,6,6-Tridecafluoro-7-pentadecene	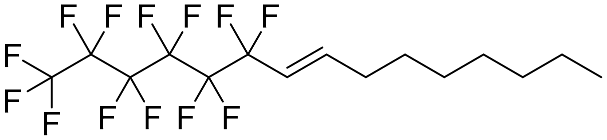
**3**	1,1,1,2,2,3,3,4,4,5,5,6,6-Tridecafluorohexadecane	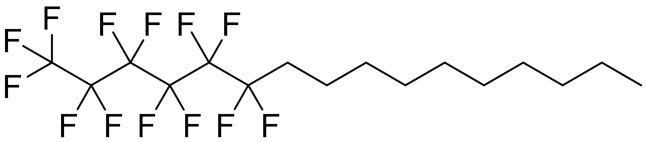
**4**	1,1,1,2,2,3,3,4,4,5,5-Undecafluorononadecane	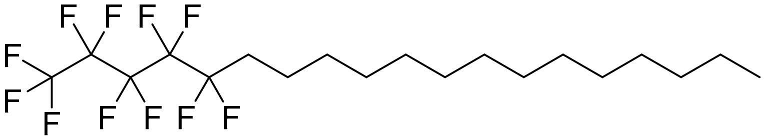
**5**	1,1,1,2,2,3,3,4,4,5,5-Undecafluoropentane	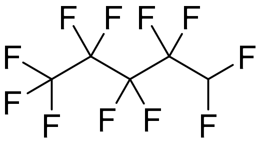
**6**	1,1,1,2,2,3,3,4,4-Nonafluorodocosane	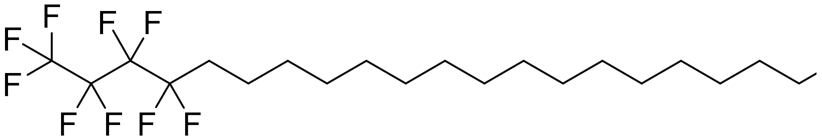
**7**	1,1,1,2,2,3,3-Heptafluorododecane	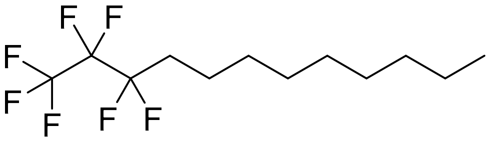
**8**	1,1,1,3,3,3-Hexafluoro-2-(trifluoromethyl)propan-2-yl heptafluorobutanoate	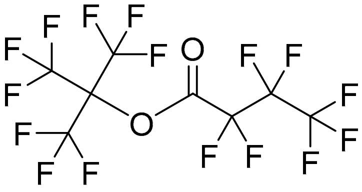
**9**	1-((3-(Dimethylamino)propyl)amino)-4,4,5,5,6,6,7,7,8,8,9,9,10,10,11,11,12,13,13,13-icosafluoro-12-(trifluoromethyl)tridecan-1-ol	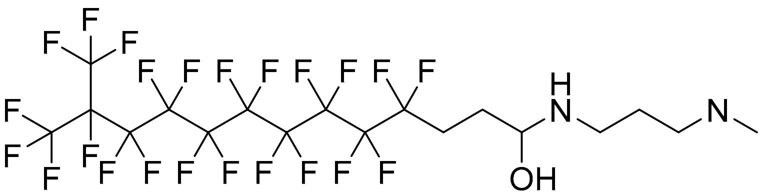
**10**	1-(2-chloro-1,1,2,3,3,3-hexafluoropropoxy)-1,1,2,3,3,3-hexafluoropropan-2-ol	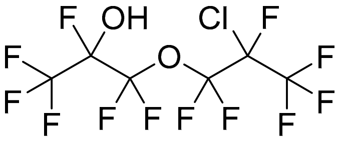

**11**	1-(Difluoromethoxy)-1,1,2,2-tetrafluoro-2-(trifluoromethoxy)ethane	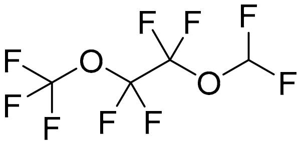
**12**	(Pentafluoroethyl)-(trifluoromethyl)cyclohexane	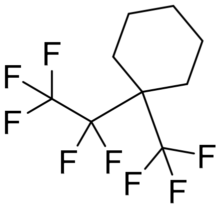
**13**	1-(Perfluoro-n-hexyl)dodecane	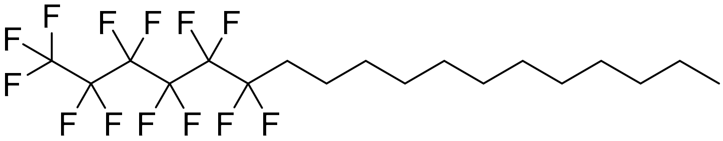
**14**	1-(Tetradec-1-en-1-yl)perfluorohexane	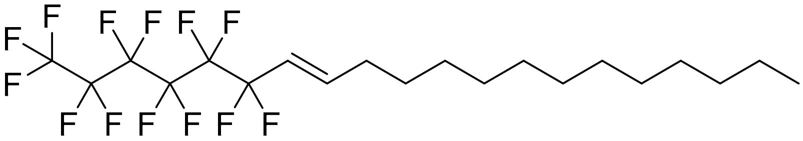
**15**	1-hydro-pentadecafluoroheptane	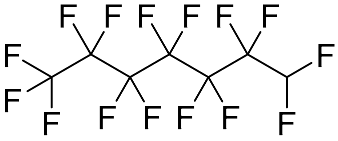
**16**	1-sec-Butyl-2-(1,1,2,2-tetrafluoroethoxy)benzene	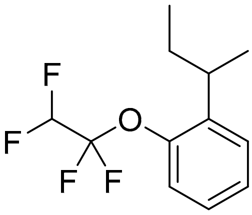
**17**	12,12,13,13,13-Pentafluorotridecanoic acid	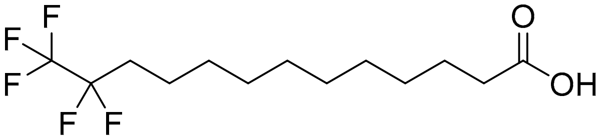
**18**	1H-Benzimidazole, 5,6-dimethyl-2-(pentafluoroethyl)-	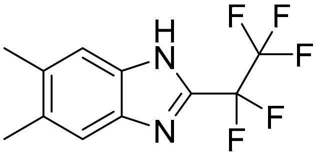
**19**	1H-Perfluorohexane	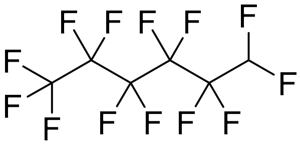
**20**	2,2,3,3,4,4,5,5,6,6,7,7,8,9,9,9-hexadecafluoro-8-(trifluoromethyl)nonanoic acid	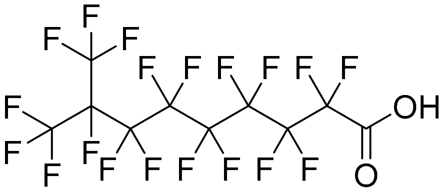
**21**	2-(3-(2-chloro-1,1,2,3,3,3-hexafluoropropoxy)-1,1,2,2,3,3-hexafluoropropoxy)-1,1,2,2-tetrafluoroethan-1-ol	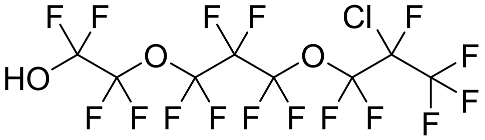

**22**	2-(Nonafluorobutyl)benzoic acid	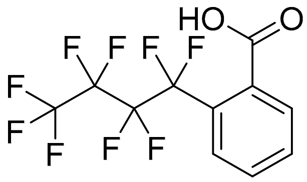
**23**	2-Ethyl-4-(1,1,1,2,3,3,3-heptafluoropropan-2-yl)-3-[(2-methylpropyl)sulfanyl]benzoic acid	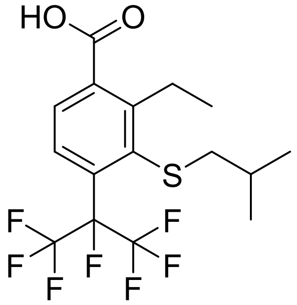
**24**	2-Vinylperfluorobutane	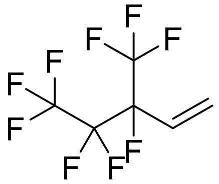
**25**	2H-Nonafluorobutane	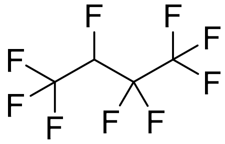
**26**	3,4,5,5,6,6,7,7,8,8,9,9,10,10,10-Pentadecafluoro-3-decen-2-one	
**27**	3-((2-chloro-1,1,2,3,3,3-hexafluoropropoxy)difluoromethoxy)-1,1,2,2,3,3-hexafluoropropan-1-ol	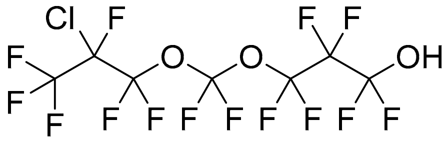
**28**	3-(12,12,13,13,14,14,15,15,15-Nonafluoropentadecyl)-1,2-benzenediol	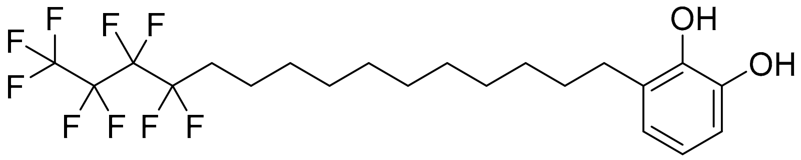
**29**	3-(3-(2-chloro-1,1,2,3,3,3-hexafluoropropoxy)-1,1,2,2,3,3-hexafluoropropoxy)-1,1,2,2,3,3-hexafluoropropan-1-ol	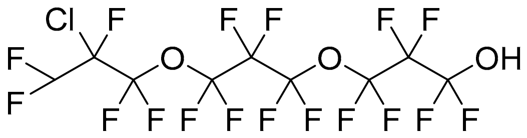
**30**	3-(Butylsulfanyl)-2-ethyl-4-(1,1,1,2,3,3,3-heptafluoropropan-2-yl)benzoic acid	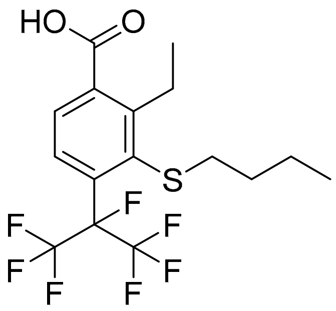

**31**	3-[Ethyl(perfluoro-1-oxopentyl)amino]-2-hydroxypropyl heptanoate	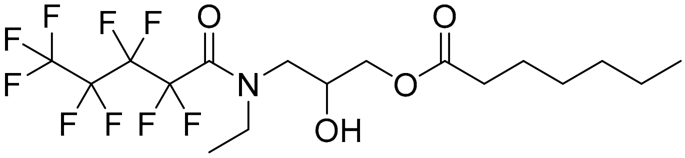
**32**	3-Fluoro-4-{(E)-[4'-(heptafluoropropyl)-4-biphenylyl]diazenyl}phenol	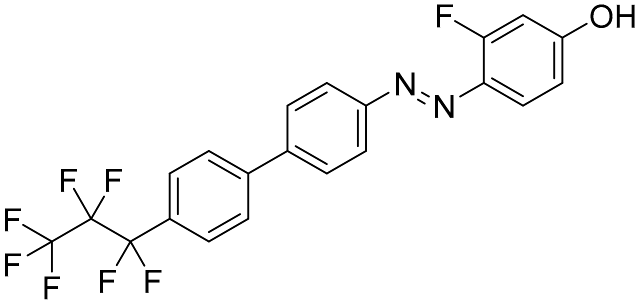
**33**	3-Pyridinecarboxamide, N-[4-(nonafluorobutoxy)phenyl]-	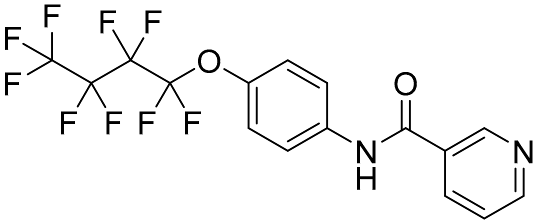
**34**	4,4-Bis(trifluoromethyl)-2H,4H-1,3-benzodioxine	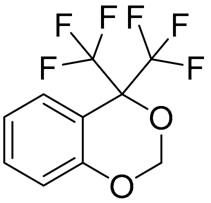
**35**	4-[Ethyl(2,2,2-trifluoroethyl)amino]-2-(trifluoromethyl)benzonitrile	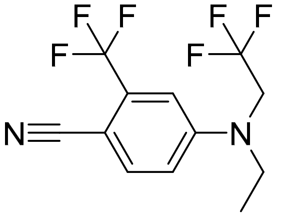
**36**	5,5,6,6,7,7,8,8,9,9,10,10,10-Tridecafluoro-1-decanol	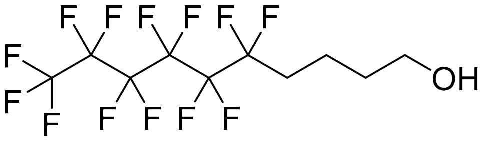
**37**	5,5,6,6,7,7,8,8,9,9,10,10,11,11,12,12,12-Heptadecafluoro-2-methyl-2-dodecanol	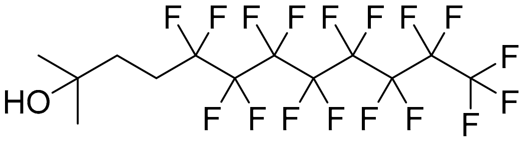
**38**	6-(1,1,1,3,3,3-Hexafluoro-2-hydroxypropan-2-yl)-3,5,5-trimethylcyclohex-2-en-1-ol	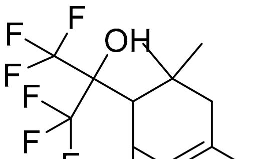
**39**	6-Chloro-1,1,2,2,3,3,4,4,5,5,6,7-dodecafluoroheptane	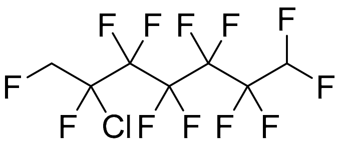

**40**	9,9,10,10,11,11,12,12,12-Nonafluorododecan-1-ol	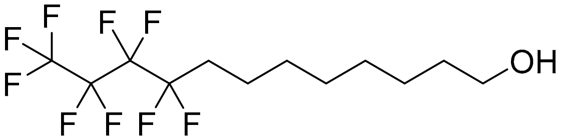
**41**	Diethyl (3,3,4,4,5,5,6,6,6-nonafluorohexyl)phosphonate	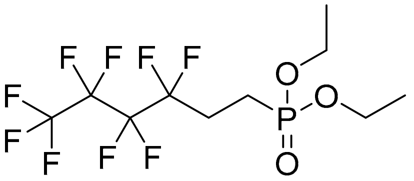
**42**	Hexadecyl 2,3,3,3-tetrafluoropropanoate	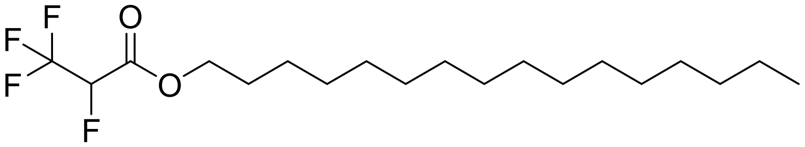
**43**	N-[3-(Dimethylamino)propyl]perfluoro-4-(methyl)cyclohexanecarboxamide N-oxide	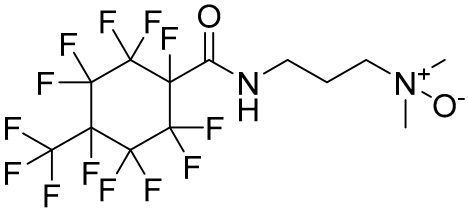
**44**	N-[3-(Dimethylamino)propyl]perfluorobutanamide	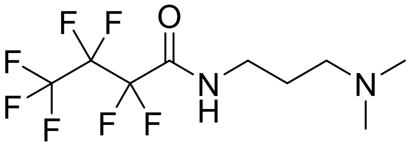
**45**	N-[3-(Dimethyloxidoamino)propyl]perfluoro-3,7-dioxaoctanamide	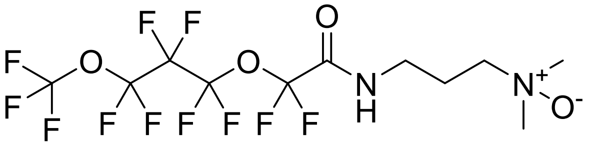
**46**	N-dihydroxyethyl amino propyl-perfluorodecane amide	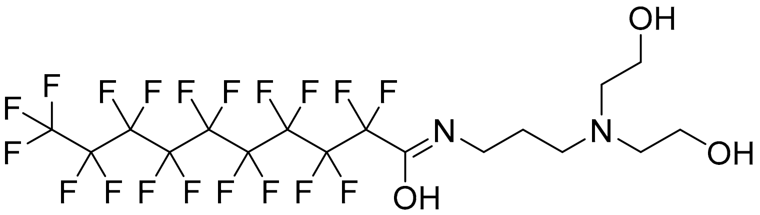
**47**	N-dimethylammoniocarboxypropyl-perfluoropropane sulfonamide	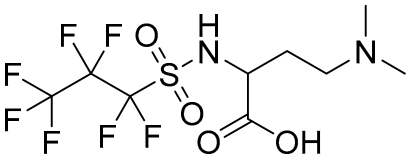
**48**	Perfluoro-2,2,3,3-tetramethyl butanoic acid	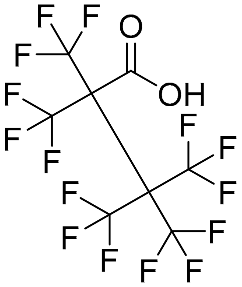
**49**	Perfluoro-6-methylheptanesulfonate	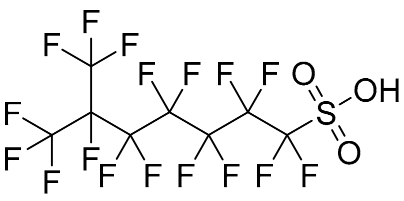

**50**	Perfluoro-n-hexanesulfonate	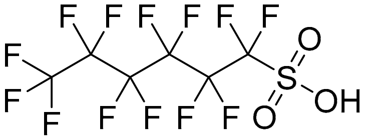
**51**	Perfluoro-n-octanesulfonate	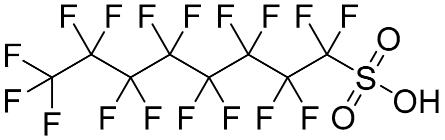
**52**	Perfluoroheptanesulphonyl fluoride	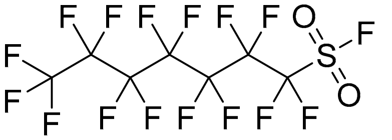
**53**	Perfluoro-n-heptanoic acid	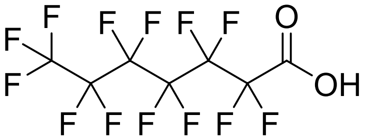
**54**	Perfluoro-n-hexanoic acid	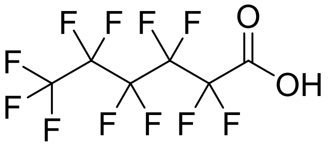
**55**	Perfluoro-n-nonanoic acid	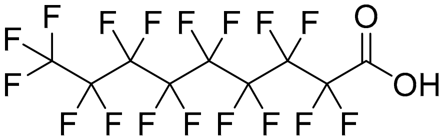
**56**	Perfluoropropyl formate	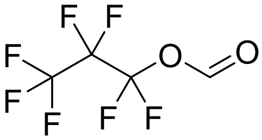
**57**	Perfluoro-n-pentanoic acid	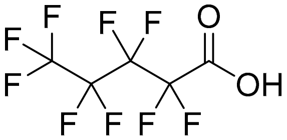
**58**	Perfluoro-n-unidecanoic acid	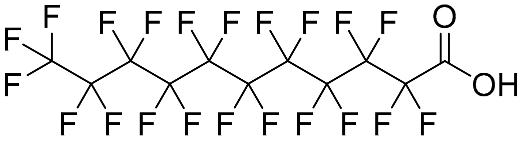
**59**	Pyrifluquinazon	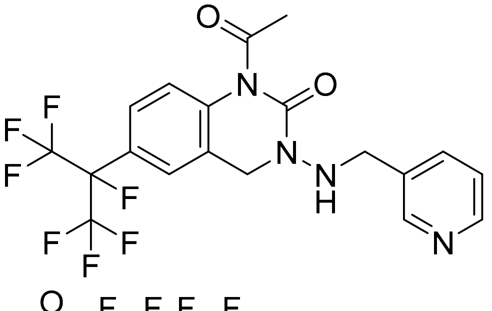
**60**	2,2,3,3,4,4,5,5,6-nonafluoro-6-oxohexanoic acid	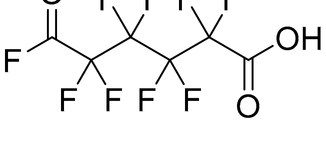
